# Eco-friendly TEMPO/laccase/O_2_ biocatalytic system for degradation of Indigo Carmine: operative conditions and laccase inactivation†

**DOI:** 10.1039/d3ra03107a

**Published:** 2023-07-11

**Authors:** Iryna O. Hordieieva, Olga V. Kushch, Tetiana O. Hordieieva, Serhii I. Sirobaba, Mykhailo O. Kompanets, Victor M. Anishchenko, Alexander N. Shendrik

**Affiliations:** a Faculty of Chemistry, Biology and Biotechnologies, Vasyl' Stus Donetsk National University Vinnytsia 21021 Ukraine i.hordieeva@donnu.edu.ua kusch.o@donnu.edu.ua; b L. M. Litvinenko Institute of Physico-Organic Chemistry and Coal Chemistry, National Academy of Sciences of Ukraine Kyiv 02660 Ukraine; c Enamine Ltd. 01103 Kyiv Ukraine

## Abstract

The biocatalytic system laccase/TEMPO/O_2_ has attracted the attention of researchers over the past two decades. A variety of applications for the system include organic synthesis, modification of cellulose, and oxidative degradation of environmental contaminants. A rigorous and predictable quantitative assessment of the change in enzymatic activity under the influence of a mediator is important for such a system. In this study, the operative conditions for carrying out a model reaction for the degradation of the synthetic dye Indigo Carmine in the presence of *Trametes versicolor* laccase/TEMPO were determined and the enzyme inactivation under the action of a mediator and substrate was studied. The long-term stability of *Trametes versicolor* laccase was assessed and the regression model of the response surface of laccase activity under the influence of TEMPO was created. It has been shown that laccase is inactivated in the presence of TEMPO, but the addition of the dye, CuSO_4_ or CuCl_2_ reduces this effect. The system under study can be used repeatedly for the Indigo Carmine decolorization, however, a gradual falling rate during the process is observed from cycle to cycle. This is due to two reasons – firstly, a decrease in the enzyme activity with each batch and secondly, the consumption of the mediator (22% within 5 days). Relatively high enzyme activity (>40%) is maintained after 73 cycles (1 portion of IC contained 25 μM) using 500 μM TEMPO and 0.12 U mL^−1^ laccase. The laccase/TEMPO system has shown its effectiveness in the treatment of artificial wastewater containing high concentrations of Indigo carmine (0.5 g L^−1^). In this case, the dye solution becomes 100% colorless within 5 hours in the presence of dye bath components and within 7.5 hours in a buffer solution.

## Abstract 2

The long-term stability of *Trametes versicolor* laccase was assessed and the operative conditions for carrying out a model reaction for the degradation of the dye Indigo Carmine in the presence of *Trametes versicolor* laccase/TEMPO were determined.

## Graphical abstract

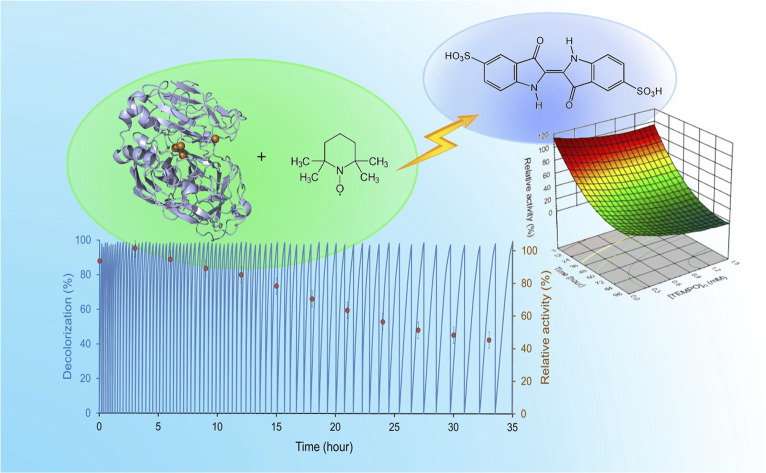

## 1 Introduction

Laccases (*p*-diphenol:oxygen oxidoreductase EC 1.10.3.2) belong to the blue-copper family of oxidases. These enzymes are considered ideal “green catalysts” because they require only molecular oxygen as the final electron acceptor for catalysis.^1^ Laccases, as nonspecific oxidative enzymes, can oxidize a wide range of substrates such as lignin, phenols, and aromatic amines.^2^ In recent years, intensive research has been directed towards the development of effective biocatalytic systems using laccases for bioremediation with the aim of oxidative degradation and detoxification of pollutants.^3–6^ But in several cases, laccases are not able to directly oxidize the substrate due to the high redox potential of the substrate or the steric inaccessibility of the enzyme active site.^7^ In this case, low molecular weight compounds, called mediators, are used in combination with laccases to indirectly oxidize organic compounds. Mediators are primary substrates of laccases and are oxidized by enzyme to active form, which in turn oxidizes organic compounds in a non-enzymatic process. There is a lot of information in the literature on mediators of various chemical nature^7–15^ such as 2,2′-azino-bis(3-ethylbenzothiazoline-6-sulfonic acid) (ABTS),^8–10^ compounds with NOH-fragment,^11–13^ 2,2,6,6-tetramethylpiperidine-1-oxyl (TEMPO),^14^ phenolic natural mediators.^15^ Each of the known mediators has its own advantages and disadvantages and operates through different mechanisms: ABTS follow an electron transfer (ET),^10^ NOH-compounds and phenolic natural mediators form active radical intermediates that oxidize substrates *via* hydrogen atom transfer,^15,16^ TEMPO-mediated oxidation proceeds by an ionic mechanism.^17–19^ The efficiency of mediators in the processes of laccase-catalyzed oxidation of organic compounds depends on the activity and stability of their intermediates.^19,20^ In addition, laccase-mediator system (LMS) often has the disadvantage that the mediators are expensive and potentially toxic. Therefore, it is necessary to carefully select the type and concentration of mediators, to study the optimal operating parameters of the oxidation process in each specific case.

The prospects for oxidative catalysis with laccase and stable radicals are extremely promising. The combination of laccase with TEMPO as mediator was first successfully used for the oxidation of benzyl alcohols.^21^ Many applications of this system have been proposed over the past twenty years, including bioremediation and detoxification of environmental pollutants,^22–24^ aerobic oxidation of non-phenolic substrates,^14,19,21,25^ selective oxidative modification of cellulose,^18,26^ sugars,^27^ cellulose nanofiber production,^28^ biocatalyzed organic synthesis, including transformation of biomass into compounds with high added value.^29–35^

**Fig. 1:**
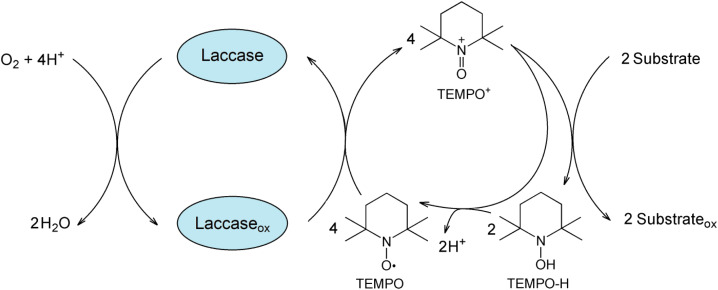
The mechanism of aerobic oxidation of organic compounds catalyzed by laccase/TEMPO/O_2_.

The mechanistic details of laccase/TEMPO catalyzed aerobic oxidation of organic compounds is still a matter of discussion.^17–19,36,37^ According with the proposed ionic mechanism,^17,18,21,36^ laccase oxidizes TEMPO with concomitant four-electron reduction of molecular oxygen to water. The resulting oxoammonium cation (TEMPO^+^) directly oxidizes organic compounds, while being reduced to *N*-hydroxyl-TEMPO (TEMPO-H) (Fig. 1). Two possible ways of TEMPO regeneration are proposed in the literature: oxidation of hydroxylamine by laccase^37^ or nonenzymatic comproportionation of oxoammonium and hydroxylamine back to two TEMPO molecules. However, as shown in the studies,^17,18^ the TEMPO regeneration by laccase is unlikely and proceeds by non-catalytic reaction of the oxoammonium cation with hydroxylamine.

The laccase/TEMPO/O_2_ system has shown a high potential in dye decolorization processes.^38–40^ Treatment of wastewater containing synthetic dyes is a serious problem due to its complex aromatic structure, toxicity of dyes and resistance to degradation.^41–43^ Most of these hazardous pollutants are discharged into wastewater with either incomplete or no treatment.^44^ Indigo Carmine (IC) is the representative indigoid dye with aromatic complex structure, widely used as coloring agent in many industries including the textile, cosmetics, food processing, biomedical research.^45^ The extensive use of IC leads to environmental contamination, causing serious damage to the health human and aquatic organisms.^46^ Numerous methods have been developed to remove IC from wastewater,^47–53^ but most of them have limitations such as expensive cost, incomplete dye decomposition or formation of toxic sludge. Ecofriendly biocatalysts based on laccases for IC removal are attractive both from economic and technical point of view.^54,55^ They can be easily combined with other water treatment technologies.

The IC decolorization is a convenient model process for evaluating the ability of enzymes to degrade persistent organic compounds (POCs). However, IC is not a typical substrate of laccases, and the complete decomposition of this dye by laccase needs the help of a mediator. In this study, commercially available laccase from *Trametes versicolor* (*T. versicolor*) was used in combination with the TEMPO mediator for the oxidative degradation of IC. *T. versicolor* laccase is the enzyme involved in lignin oxidation, modification or degradation. This laccase is industrially important enzyme due to its high redox potential (785 mV)^56^ and high activity towards a wide range of substrates. In recent years, *T. versicolor* fungi laccase has gained much attention due to its removal of ecopollutants by enzymatic degradation and adsorption.^57^

Despite considerable attention to the processes of oxidation of organic compounds in the presence of the laccase/TEMPO/O_2_ biocatalytic system, there is not enough information on the kinetics of oxidative degradation of dyes using this LMS, as well as on the stability of *T. versicolor* laccase under the TEMPO action. In this paper, we also considered the possibility of multiple use of the system for the IC treatment.

## 2 Materials and methods

### 2.1 Materials

All chemicals including laccase from *T. versicolor* (1.07 U mg^−1^), TEMPO, IC, CuSO_4_·5H_2_O, CuCl_2_·2H_2_O and Cu(NO_3_)_2_·3H_2_O were purchased from Sigma-Aldrich and used as received.

CuSO_4_, CuCl_2_ and Cu(NO_3_)_2_ solutions were prepared in the bidistilled water using CuSO_4_·5H_2_O, CuCl_2_·2H_2_O and Cu(NO_3_)_2_·3H_2_O respectively. An aliquot (1 mL) of the resulting solution was titrated with 0.05 M EDTA solution at pH 7.0, which was maintained by adding 0.1 M solutions of NH_3_·H_2_O and NH_4_Cl. Murexide was used as indicator. The equivalence point was fixed by the color transition from yellow to purple. The established concentration of CuSO_4_, CuCl_2_ and Cu(NO_3_)_2_ solutions were 0.32 M, 0.27 M and 0.34 M respectively (error 1%).

### 2.2 Assay for laccase activity

Laccase activity was measured spectrophotometrically by the oxidation of ABTS using Analytic Jena SPECORD 50 UV-Vis spectrophotometer. The reaction mixture contained 0.1 mL of ABTS (1 mM), 2.3 mL of citrate-phosphate buffer (McIlvaine buffer (0.1 M citric acid and 0.2 M disodium phosphate)) (0.1 M, pH 4.5), and 0.6 mL *T. versicolor* laccase (120 μg mL^−1^, 0.12 U mL^−1^) into 3.0 mL quartz cuvette. The solutions were kept at 35 °C for 60 s and the resulting absorbances were measured at 420 nm (*ε* = 3.6 × 10^4^ M^−1^ cm^−1^).^58^ Determination of enzyme activity in the presence of TEMPO conducted into 3.0 mL quartz cuvette containing 0.1 mL of ABTS (0.25 mM), 2.75 mL of citrate-phosphate buffer at pH 4.5, and 0.15 mL reaction mixture. Enzyme activity was expressed in international units. One unit of activity is defined as the amount of laccase oxidizing 1 μmol of substrate per min. Laccase activity was calculated using eqn (1):^59^1
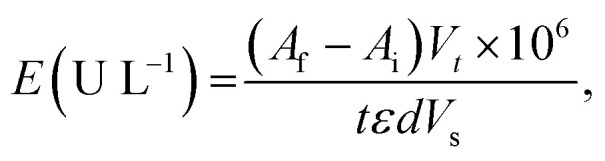
where *E* is the activity of enzyme (U L^−1^); *A*_f_ is a final absorbance; *A*_i_ is an initial absorbance; *V*_t_ is total volume of the reaction system into cuvette (mL); *V*_s_ is sample volume of enzyme (mL); *t* is reaction time (min); *ε* is molar extinction coefficient (M^−1^ cm^−1^); *d* is an optical path (1 cm); 10^6^ is correction factor to convert mol L^−1^ into μmol L^−1^. The assay was carried out at the concentration of ABTS in the region of saturation of the enzyme with the substrate (see Fig. S1 and S2†). In the investigation used solutions of *T. versicolor* laccase with activity 6 × 10^−3^ U mL^−1^ and 0.12 U mL^−1^ respectively.

### 2.3 IC decolorization experiments

IC decolorization was studied by registering the decrease in solution absorbance at the maximum wavelength of dye (614 nm). Reaction mixture contained 0.6 mL *T. versicolor* laccase (0.12 U mL^−1^), 0.1 mL dye (25 μM), 0.4 mL TEMPO (240 μM) and 1.9 mL of citrate-phosphate buffer (0.1 M) at pH 4.5 and at 35 °C into 3.0 mL quartz cuvette. The decolorization efficiency or percentage of color removal was determined according to eqn (2):2
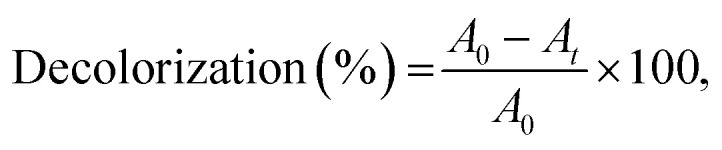
where *A*_0_ and *A*_*t*_ denote the initial absorbance and after treatment at a certain time *t*. Control samples were run in parallel with laccase or mediator alone. Doubly distilled water was used to prepare solutions. For each assay duplicate runs were made.

### 2.4 Effect of pH on the IC decolorization and oxidation of hydroquinone and ABTS by laccase

The effect of pH on the IC decolorization was studied by treating 0.1 mL dye (25 μM) with 0.1 mL *T. versicolor* laccase (0.12 U mL^−1^) in the presence of 0.1 mL TEMPO (500 μM) and 2.7 mL of citrate-phosphate buffer (0.1 M) at different pH: 2.0, 2.5, 3.0, 3.5, 4.0, 4.5, 5.0, 5.5, 6.0, 6.5, 7.0, 7.5, 8.0, 8.5 in 3.0 mL quartz cuvette. The reaction mixtures were kept at 35 °C for 30 minutes and the resulting absorbances were measured at *λ*_max_ of IC.

The effect of pH on the hydroquinone (HQ) or ABTS oxidation was studied by treating 0.1 mL of substrate (500 μM and 1.0 mM in cuvette for HQ and ABTS respectively) with 0.1 mL *T. versicolor* laccase (0.12 U mL^−1^) in the presence of 2.8 mL of citrate-phosphate buffer (0.1 M) at different pH: 2.0, 2.5, 3.0, 3.5, 4.0, 4.5, 5.0, 5.5, 6.0, 6.5, 7.0, 7.5, 8.0, 8.5 in 3.0 mL quartz cuvette. The reaction mixtures were kept at 35 °C and the resulting absorbances were measured at *λ*_max_ of HQ (291 nm, *ε* = 3240 M^−1^ cm^−1^) or ABTS˙^+^ (420 nm).

### 2.5 Effect of TEMPO concentration on the IC decolorization

The effect of the mediator concentration on the IC decolorization was studied using of 0.1 mL IC (25 μM) in the presence of TEMPO in the concentrations ranged from 60 to 1250 μM by step 60 μM with 0.6 mL *T. versicolor* laccase (0.12 U mL^−1^) in 3.0 mL quartz cuvette in the citrate-phosphate buffer at pH 4.5 and 35 °C. All the experiments were performed in triplicate.

### 2.6 Reusability of the laccase-mediator system

The reusability of the laccase/TEMPO system was examined using 0.6 mL *T. versicolor* laccase (0.12 U mL^−1^) and 0.4 mL TEMPO (500 μM) for successive IC decolorization for 85 cycles (35 h) adding 10 μL of dye (25 μM) in each of them. The total amount of added dye was 2.12 mM (1.0 g L^−1^). The reaction was run until to complete IC decolorization was achieved. Experiments were set up as described in Section 2.1, adding only IC solutions in the water when starting a new batch.

### 2.7 Determination of products of the IC oxidation

IC oxidation products after complete decolorization of dye were determined by HPLC and identified by comparing their retention times and UV-Vis spectra to those of known compounds. The reaction mixture consisted of 0.5 g L^−1^ of IC, 0.12 U mL^−1^*T. versicolor* laccase and 500 μM TEMPO in the citrate-phosphate buffer at pH 4.5 at 35 °C. Composition of reaction mixtures were monitored by RP-HPLC using an Agilent 1100 system with a diode array detector. Separations were carried out on the chromatographic column ZORBAX SB-C18 (250 mm × 4.6 mm, 5 μm). The following gradient composition used for each analysis: 0–3 min – 90% A + 10% B at the flow rate 0.5 mL min^−1^, 8 min – 60% A + 40% B at the flow rate increased to 1 mL min^−1^, 15 min – 0% A + 100% B at the flow rate increased to 1.5 mL min^−1^, 20 min – 0% A + 100% B at the flow rate 1.5 mL min^−1^, where A is water (0.05 M H_3_PO_4_) and B is methanol. The injection volume was 5 μL, column temperature was 35 °C. Detection was performed at the wavelengths of 206 nm, 230 nm, 242 nm, and 372 nm.^12^

### 2.8 Decolorization of the simulated textile effluent

The composition of the simulated textile effluent was based on instructions of the manufacturer Bezema AG (Montlingen, Switzerland) for acid dyes.^60^ It consisted of 0.5 g L^−1^ of IC and 50 g L^−1^ of Na_2_SO_4_, and 1.0 mL L^−1^ of 80% CH_3_COOH in bidistilled water. The pH was adjusted to 4.5 with HCl. The solutions were incubated in volume 50 mL at 35 °C. The reaction mixture contained 6.2 mg *T. versicolor* laccase (0.12 U mL^−1^), 500 μM TEMPO and 25 mg IC (0.5 g L^−1^) in the reaction mixtures. Aliquots (0.1 mL) were diluted in 2.9 mL in simulated textile effluent and registrated absorbance at the maximum wavelength of IC (614 nm) in 3.0 mL quartz cuvette. Spectral locations of the wavelengths of maximum absorption of IC, TEMPO and ABTS^+^˙ neither overlaps nor interfere with the activity assay. No magnetic stirring was used. The decolorization efficiency was determined according to eqn (2).

### 2.9 Effect of TEMPO on laccase activity

Laccase activity was measured at several concentrations of TEMPO (0, 0.1, 0.3, 0.5, 0.8, 1.0, 1.3, 1.5 mM) both in the presence and in the absence of IC in citrate-phosphate buffer at pH 4.5 and 35 °C. The initial laccase activity was 0.12 U mL^−1^. The experiments were carried out in 10 mL Erlenmeyer flasks. Samples were taken at selected reaction times: 0, 3, 6, 9, 12, 24, 36, 48, 60, 72, 84, 96 h and subsequently diluted (1 : 20) using citrate-phosphate buffer at pH 4.5. The dilution avoided rapid oxidation of ABTS by TEMPO^+^. Aliquots (0.15 mL) were diluted in 2.8 mL of citrate-phosphate buffer pH 4.5, in 3.0 mL quartz cuvette, incubated at 35 °C for 5 minutes, added 50 μL of ABTS (250 μM) and analyzed the residual activity using the procedure described above in Section 2.2. The control experiment was carried out without TEMPO in the reaction mixture under similar conditions.

Comparing obtained results were use eqn (3) for relative activity:3
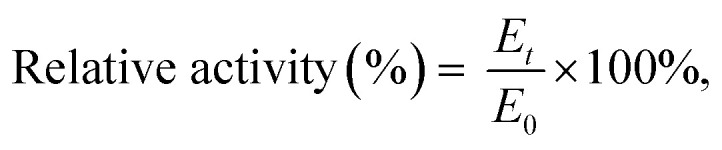
where *E*_*t*_ and *E*_0_ are the current activity of *T. versicolor* laccase in the reaction mixture and initial activity of enzyme in the citrate-phosphate buffer at pH 4.5 (U mL^−1^) respectively.

### 2.10 Effect of IC on laccase activity

The assay reaction mixture (50 mL) contained 0.5 g L^−1^ or 2.0 g L^−1^ IC (25 mg or 100 mg) respectively, 6.2 mg *T. versicolor* laccase (0.12 U mL^−1^) both with and without TEMPO (500 μM). Reaction mixtures were incubated at 35 °C and aliquots (0.15 mL) were sampled at different incubation periods: 0, 3, 6, 9, 12, 24, 36, 48, 60, 72 h. Aliquots were diluted in 2.8 mL citrate-phosphate buffer at pH 4.5 in 3.0 mL quartz cuvette and incubated at 35 °C for 5 minutes, added 50 μL of ABTS (250 μM) and analyzed the residual activity using the procedure described above in Section 2.2. Absorption bands of IC, TEMPO and ABTS^+^˙ have neither overlap nor interfere with the activity analysis. Control experiment was provided in the absence of IC and TEMPO in similar conditions. The relative activity was determined according to eqn (3).

### 2.11 Effect of CuCl_2_, Cu(NO_3_)_2_ and CuSO_4_ on the laccase activity

Laccase activity in the presence of CuSO_4_, CuCl_2_ or Cu(NO_3_)_2_ was determined by monitoring the oxidation of ABTS at 420 nm. The reaction mixture (50 mL) included several CuSO_4_, CuCl_2_ or Cu(NO_3_)_2_ concentrations (0.5, 1, 5, 10 mM), 6.2 mg *T. versicolor* laccase (0.12 U mL^−1^) both with and without TEMPO (500 μM). Reaction mixtures were incubated at 35 °C and aliquots 2.8 mL without TEMPO and 0.15 mL in presence of TEMPO were sampled at different incubation periods: 0, 3, 6, 9, 12, 24, 36, 48, 60, 72 h. Aliquots 0.15 mL were diluted in 2.8 mL citrate-phosphate buffer at pH 4.5 in 3.0 mL quartz cuvette, incubated at 35 °C for 5 minutes, added 50 μL of ABTS (250 μM). 0.1 mL of ABTS (1 mM) was added in the aliquot without TEMPO (2.9 mL) and analyzed the residual activity using the procedure described above in Section 2.2. Control experiment was provided neither CuSO_4_, CuCl_2_, Cu(NO_3_)_2_ nor TEMPO in the reaction mixture under similar conditions. Spectral locations of the wavelengths of maximum absorption of CuSO_4_, CuCl_2_, Cu(NO_3_)_2_, TEMPO and ABTS^+^˙ neither overlaps nor interfere with the activity assay.

## 3 Results and discussion

### 3.1 IC decolorization in the presence of laccase/TEMPO system

**Fig. 2:**
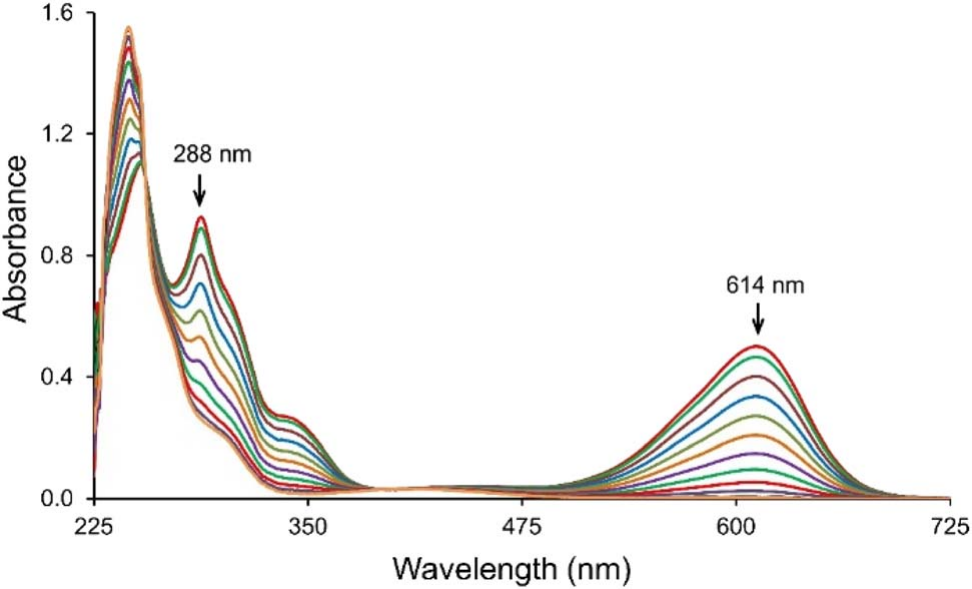
UV-Vis absorption spectra of the IC consumption in the presence of laccase/TEMPO. [IC]_0_ = 25 μM, [laccase]_0_ = 0.12 U mL^−1^ and [TEMPO]_0_ = 240 μM in the citrate-phosphate buffer pH 4.5 at 35 °C.

The IC transformation was analyzed by monitoring the decrease in absorption band at 614 nm. Corresponding spectra of the IC solutions during treatment with the *T. versicolor* laccase/TEMPO system is shown in Fig. 2. The intensive blue color of IC solution turned to a pale-yellow during the reaction and the color removal was achieved without the stirring of solution. The results suggest the cleavage of the double bond of dye chromophoric structure and new compounds formation. Laccase/TEMPO system provided complete decolorization of IC solution in about 1000 s in the presence of mediator. Decolorization of IC in the presence only mediator or laccase in blank experiments was not observed during the same time.

### 3.2 Effect of pH

**Fig. 3:**
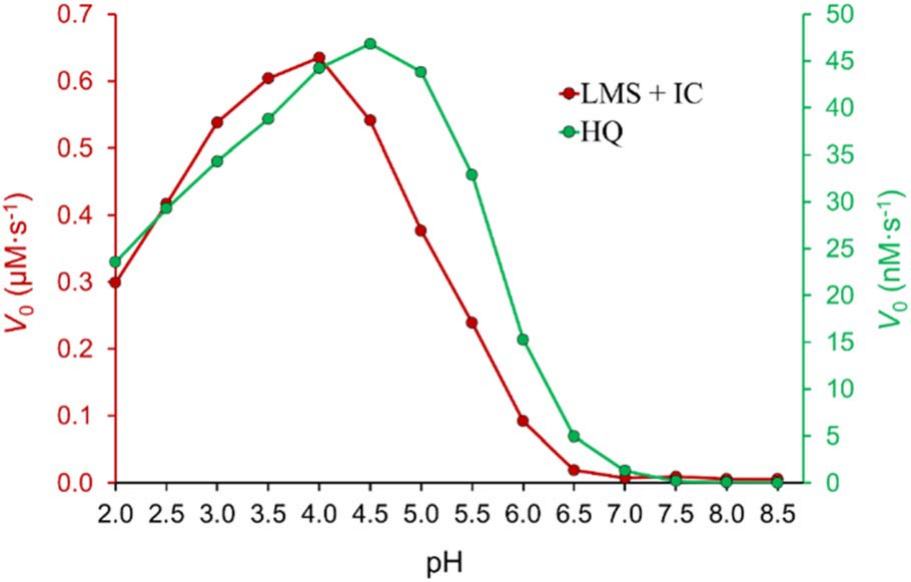
Effect of pH on the initial rate of HQ oxidation by laccase and IC by LMS. [IC]_0_ = 25 μM, [laccase]_0_ = 0.12 U mL^−1^, [TEMPO]_0_ = 500 μM, and [HQ]_0_ = 500 μM in the citrate-phosphate buffer at 35 °C.

The influence of pH on the process of IC decolorization in the presence of the laccase/TEMPO system was studied in the pH range of 2.0–8.5 with other fixed parameters. The dependence has a bell-shaped form (Fig. 3), which is characteristic of enzymatic reactions involving laccases. The observed pH profile reflects the combined result of pH-dependent transformations of the enzyme, mediator, and dye. The pH optimum depends both on the type of laccase,^61^ and the chemical nature of the substrate.^12,62^ It is known that pH affects the conformation and activity of the enzyme, the stability of the mediator, and its ability to bind to the active site of laccase.^61,63^ The highest decolorization rate was achieved under acidic conditions in the pH range from 4.0 to 5.0, the optimal pH for *T. versicolor* laccase activity in citrate-phosphate buffer.^64^ As can be seen from Fig. 3 biocatalytic system is ineffective at 3.5 > pH > 5.5. Saoudi *et al.*^63^ showed that an excess either H^+^ or OH^−^ ions destroy hydrogen bonds and disulfide bridges in *T. versicolor* laccase, causing a decrease in the functional ability of the enzyme. In addition, the enzyme is inhibited by hydroxyl ions with an increasing pH (involving T2 Cu in laccase).^61^ It should be noted that TEMPO-mediated aerobic oxidation is a complex multi-stage process. The pH of the medium affects both activity of laccase, and interconversions of TEMPO, TEMPO^+^, and TEMPO-H. It is reported that reactions involving TEMPO and its reactive forms are sensitive to the pH of the medium.^18^ One-electron oxidation of TEMPO with laccase in the presence of O_2_ to the oxoammonium ion TEMPO^+^ is effective in an acidic medium.^18^ Increasing pH to 6.8 is preferable for the comproportionation reaction of TEMPO^+^ with TEMPO-H to form TEMPO.^65^ However, as mentioned above, laccase activity sharply decreases with increasing pH. In general, the observed dependence (Fig. 3) is very similar to the pH profile of *T. versicolor* laccase activity obtained when used hydrohinone (Fig. 3) syrinaldehyde,^64^ TEMPO^66^ as substrates. Obtained dependence of pH on *V*_0_ of the process of IC decolorization indicates that pH primarily affects the enzymatic reaction, and not the second cycle (chemical reaction) of the process under study.

### 3.3 Effect of initial mediator concentration

**Fig. 4:**
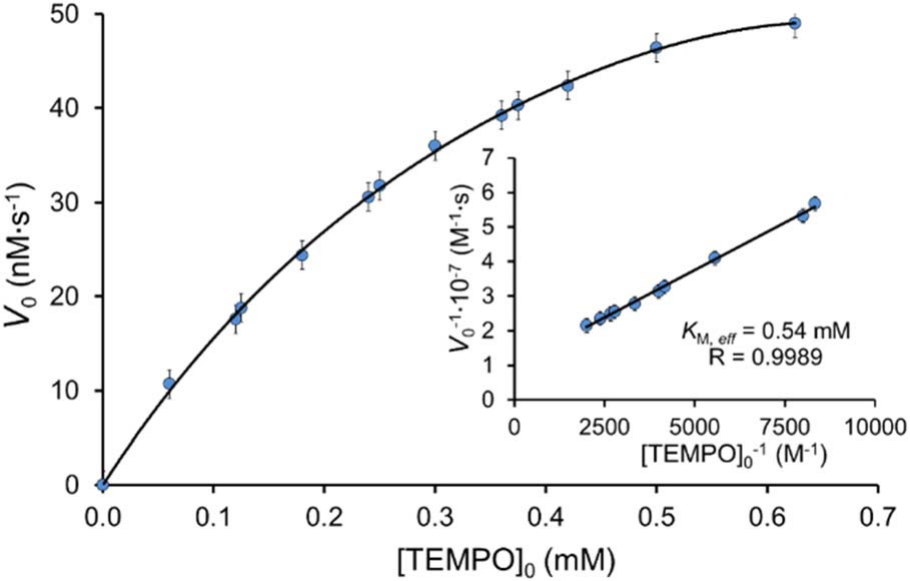
Effect of TEMPO concentration on the initial rate of IC oxidation. [IC]_0_ = 25 μM and [laccase]_0_ = 0.12 U mL^−1^ in the citrate-phosphate buffer at pH 4.5 at 35 °C.

Fig. 4 shows the dependence of the reaction rate of IC decolorization on the TEMPO concentration from 60 μM to 1250 μM in the presence of laccase (0.12 U mL^−1^). The plot has saturation region and obeys Michaelis–Menten kinetics. The Lineweaver–Burk plot was used to determine kinetics parameters: *K*_M,eff_ (0.54 ± 0.05) mM and *V*_max,eff_ (99 ± 5) nM s^−1^. Previously, the elementary step of TEMPO enzymatic oxidation has been directly studied for TEMPO and laccase from *T. versicolor*,^17,18,25,36,37,65,67,68^ and Michaelis constants *K*_M_ were determined 1.8 mM,^68^ 1.314 mM,^25^ 0.393 mM.^37^ Even though we studied the general process of IC decolorization instead of the oxidation reaction of TEMPO by laccase, we obtained a very close value *K*_M,eff_.

### 3.4 Reusability of the laccase-mediator system

**Fig. 5:**
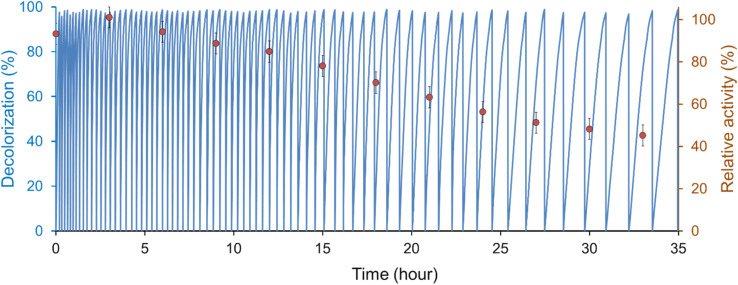
Decolorization of IC by *T. versicolor* laccase/TEMPO in a continuous recycling mode. Reaction mixtures containing [laccase]_0_ = 0.12 U mL^−1^, [TEMPO]_0_ = 500 μM in the citrate-phosphate buffer at pH 4.5 were incubated consecutively with 25 μM IC at 35 °C. Relativity activity of laccase (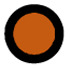).

The reusability of *T. versicolor* laccase/TEMPO to decolorize IC was investigated for 73 cycles. Decolorization was carried out by sequential addition of the dye to the reaction mixture. Each cycle was continued until the complete disappearance of the dye color, and then another dose of 25 μM (11.7 ± 0.1 mg L^−1^) IC was added to the solution to start the next cycle without adding enzyme or mediator. The amount of used TEMPO was 21.5 mol% for treatment 0.85 ± 0.05 g L^−1^ of IC at laccase activity 0.12 U mL^−1^ (Fig. 5). At the same time, the catalytic system retained the ability to effectively bleach the dye after 73 cycles.

**Fig. 6:**
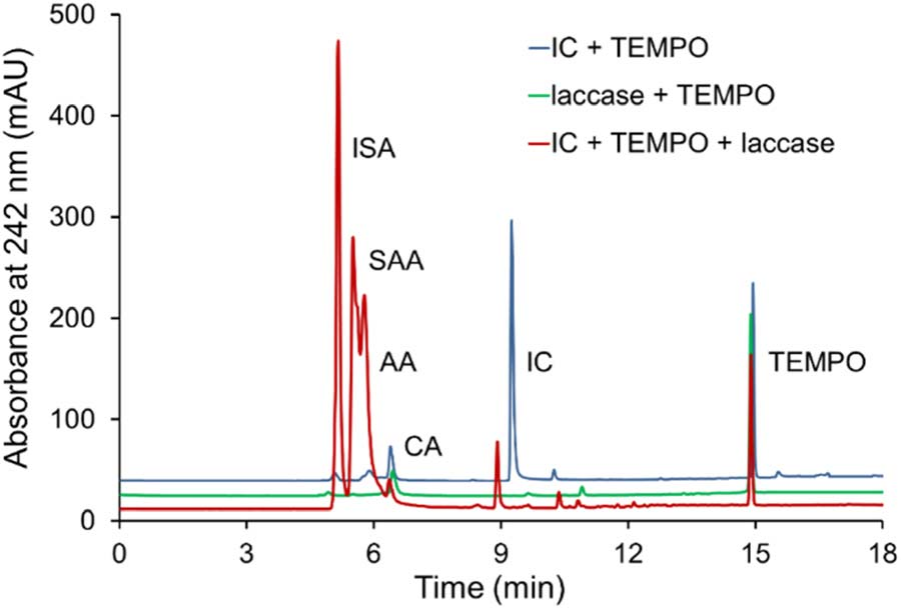
Chromatogram of IC degradation products sample (100 μL) containing decolorization mixture of IC by laccase and TEMPO; laccase and TEMPO; IC and TEMPO in the citrate-phosphate buffer at pH 4.5 at 35 °C. [IC]_0_ = 0.5 g L^−1^, [laccase]_0_ = 0.12 U mL^−1^, [TEMPO]_0_ = 500 μM.

Complete decolorization of the dye (98%) was achieved within 12.5 min after the first IC addition, but the reaction time gradually increased from cycle to cycle and reached 85 min in the 73rd cycle. In this case, the decolorization rate (*V*_0_) decreased by 10 from (3.3 ± 0.2) × 10^−8^ M s^−1^ (*t* = 0, cycle 1) to (3.5 ± 0.3) × 10^−9^ M s^−1^ (*t* = 33 h, cycle 73). A gradual decrease in the reaction rate may be associated with a fall in enzyme activity and/or the inefficient consumption of mediator in the system. HPLC analysis of IC decomposition products (Fig. 6) using LMS showed that the TEMPO concentration decreased by 22.5 ± 1.0% compared to initial one after 5 days of the reaction solution incubation. Arends *et al.*^19^ showed that the oxoammonium cation is not stable in an acidic buffer, and its decomposition can occur during laccase catalyzed oxidation. On the other hand, it is known, that *T. versicolor* laccase is unstable in the presence of TEMPO.^19^ These main reasons for the slowing down of the decolorization process agree with our results on changes in laccase activity during IC decolorization. Fig. 5 shows that the residual activity of laccase was 42% after 35 h IC decolorization with sequential addition of a dye. The laccase inactivation mechanism by TEMPO is still not well understood, but the deactivation of laccase presumably occurs due to the oxidation of alcohol groups of amino acid residues or glycosyl fragments of the enzyme by reactive oxoammonium particles.^19^

### 3.5 Analysis of products of the IC degradation

The oxidation products of IC by *T. versicolor* laccase and TEMPO were analyzed by HPLC and identified by comparing their retention times and UV-Vis spectra of known compounds. The disappearance of the dye peaks and appearance of the new peaks during the course of reaction indicates the dye degradation and formation of new metabolites. The chromatogram of IC oxidation and the UV absorption spectra (Fig. 6) show clearly the formation of isatin-5-sulfonic acid (ISA) (5.2 min), 4-sulfo-3-anthranilic acid (SAA) (5.5 min) and anthranilic acid (AA) (5.7 min) with the corresponding disappearance of the IC peak (9.4 min). The peaks at 6.4 min and 14.9 min refer to citric acid (CA) (component of buffer) and TEMPO respectively. The chromatogram also contains traces of unknown UV-active molecules with retention times *ca.* 9.0 min. The detection peaks were performed at three different wavelengths to test the proposed path.

The mechanism of IC oxidation to form ISA, SAA and AA is known and described (Scheme S1†).^11,69^ It can be assumed that the first stage is the cleavage of the double bond of ISA, the next stage is decarboxylation of ISA to SAA with subsequent elimination of the sulfo-group and the formation of AA. Isatins are known to be biologically active compounds and important precursor molecules in organic synthesis. The formation of isatin derivatives during the oxidation of the IC dye may lead to alternative methods for the synthesis of various drugs based on them.^70^

### 3.6 Decolorization of the simulated textile effluent

The dye wastewater discharged from the textile industry usually contains high levels of various inorganic salts, detergents, and other additives. The ability of LMS to maintain stability in real systems is very important for their effective use in wastewater treatment. The presence of salts and high dye concentration can inhibit the enzyme action.

**Fig. 7:**
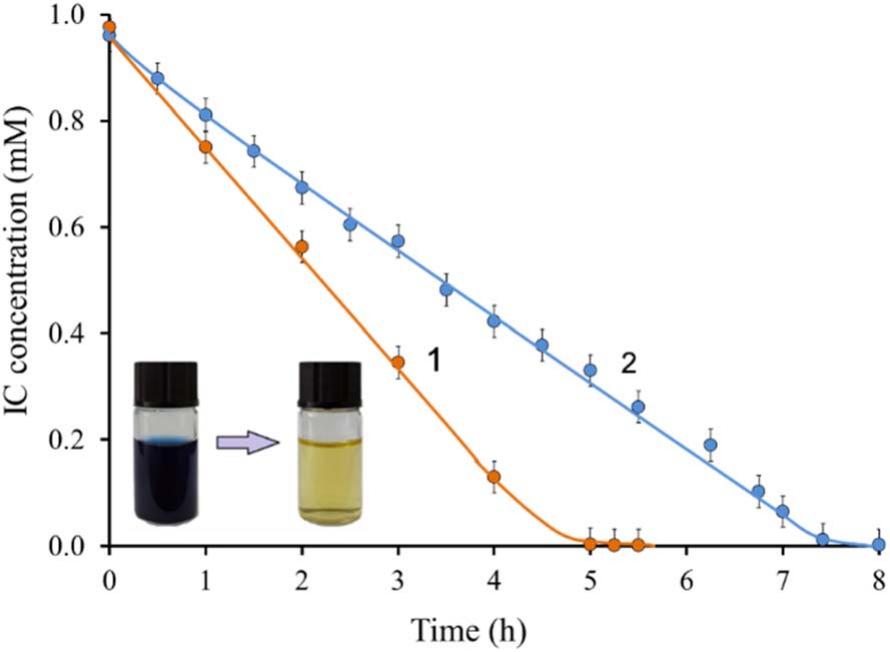
The kinetic curve of IC consumption in the presence of laccase/TEMPO system by adding Na_2_SO_4_ i CH_3_COOH (1) and in the citric-phosphate buffer (2) at pH 4.5. [Laccase]_0_ = 0.12 U mL^−1^, [TEMPO]_0_ = 500 μM, [IC]_0_ = 0.5 g L^−1^, 35 °C.

Experiments were carried out with simulated textile drains to test the effectiveness of the method for a more complex real system. As shown in Fig. 7, the decolorization rate of IC in the presence of salts and high dye concentration (0.5 g L^−1^) is even faster than in a buffer solution. After 5 hours, complete IC decolorization is observed compared to 7.5 hours in buffer. These results are consistent with previous studies, which showed that the rates of degradation processes of pollutants catalyzed by laccases in real wastewater were higher than in buffer solutions.^71^ Thus, the laccase/TEMPO system showed high efficiency, stability, and tolerance to the components of the reaction mixture. On Fig. 7 shows IC decolorization kinetic curves for simulated textile stocks (1) and buffer solution (2). Such a difference in the behavior of the laccase/TEMPO system can be associated with the influence of salts present in the buffer solution and simulated effluents.

### 3.7 Effect of TEMPO on laccase activity

**Fig. 8:**
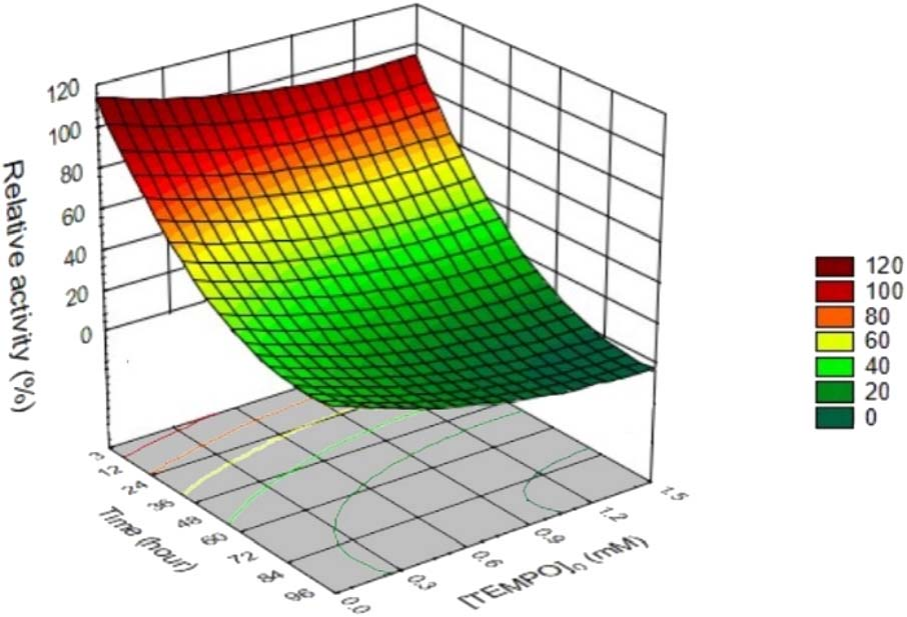
Response surface plot and contour plot of laccase relative activity from the initial TEMPO concentration and time.

To understand the effect of TEMPO, we assayed the laccase activity within 96 h at a constant initial laccase concentration of 0.12 U mL^−1^ both in the absence and in the presence of different concentrations of mediator. In total, as a result of independent experiments, 96 points were obtained (Table S1†), which were used to plot the response surface of laccase activity from initial concentration of TEMPO and reaction time (Fig. 8).

Fig. 8 demonstrates that approximately 31.6% of the initial laccase activity remained after 4 days of incubation in citrate-phosphate buffer at pH 4.5 in the absence of TEMPO. Earlier studies showed^72,73^ that the laccase from *T. versicolor* is inhibited by the citric acid. Authors believe that this might be due to the formation of complexes with the copper ions as well as by changes of the medium acidity. The addition of different TEMPO concentrations sharply reduced laccase activity. In the presence of 1.5 mM TEMPO, the laccase was completely deactivated already within 96 h. A decrease in the mediator concentration to 0.1 mM led to enzyme deactivation with the residual activity of 13.0%. Previously the inactivation of *T. versicolor* laccase under TEMPO was studied^24,28,38,74–76^ and decrease in the enzyme activity was observed. However, all studies were carried out under different conditions. The authors of works^38,74–76^ determined laccase activity only at one concentration of TEMPO. Jiang *et al.*^28^ monitored the enzyme activity in the presence of kraft cellulose, and Kobakhidze *et al.*^24^ analyzed the activity of immobilized laccase in the presence of TEMPO. In this work, 3D modeling made it possible to obtain a quantitative assessment of the change in enzymatic activity at eight TEMPO concentrations (from 0 to 1.5 mM) over time.

### 3.8 Effect of IC on laccase activity

**Fig. 9:**
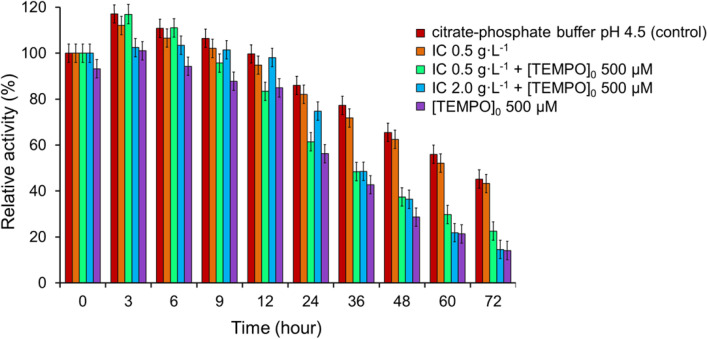
Impact of IC and TEMPO on the laccase activity. The error bars represent the standard deviation of triplicate samples.

Then we examined the effect of IC on laccase activity for 3 days. As can be seen from Fig. 9 laccase activity increased in a buffer solution to a maximum value on 3 h (117.1%), as well as at high concentrations of IC (0.5 g L^−1^, but not 2.0 g L^−1^) both in the presence (116.8%), and in the absence of a mediator (112.1%). Stimulation of laccase activity may be the result of physiological changes in laccase due to the toxicity of the compounds, as previously demonstrated for various dyes^77^ and other xenobiotics.^78^ A decrease in enzyme activity was observed for all studied systems after 12 h. The data of Fig. 9 show that the dye in the absence of TEMPO weakly inactivated laccase since the relative activity in the presence of IC approximately coincided with its values in the buffer solution. The greatest decrease enzyme activity was observed in the presence of only the mediator, while the residual activity was 56.3% after 24 h and 14.1% after 72 h of the incubation period. The addition of IC (0.5 g L^−1^) in the presence of TEMPO resulted in a slight but statistically significant delay in the decline in enzyme activity: the residual activity of the enzyme reached 61.4% after 24 hours and 22.5% after 72 h. The positive effect of IC is even more significant at concentration of 2.0 g L^−1^: following 6, 9, 12, 24 h of incubation, the residual activity of the enzyme was 103.4, 101.5, 98.1 and 74.8%, however, after 36 h, the fall in laccase activity accelerated and reached 14.6% at 72 h.

The laccase-generated oxoammonium cation TEMPO^+^ appears to be responsible for the enzyme inactivation because IC slows down the inactivation of laccase by TEMPO. TEMPO^+^ is active form of the mediator that oxidizes IC in a non-enzymatic catalytic cycle (Fig. 1), but it also reacts with laccase, thereby inactivating the enzyme. When a substrate is added, TEMPO^+^ is rapidly consumed in the oxidative process, so laccase inactivation slows down. A mechanism involving two competing processes previously has been described for TEMPO^19^ and other mediators.^79,80^ The authors showed that high concentrations of mediators maintain high reaction rates, but also lead to rapid enzyme inactivation. It is important to limit the mediator concentration in reaction systems in order to maintain the catalytic stability of laccase in the long term for use in continuous processes. For research, we chose the optimal mediator concentration of 500 μM, at which a high rate of the process is observed (see Fig. 5) and relatively low inhibition of laccase (Fig. 9). TEMPO (500 μM) and laccase (1.5 U mL^−1^) were successfully used by the authors of the works for the oxidation of phenol.^76^ Feng *et al.* reported a mediated *T. versicolor*/TEMPO the transformation of β-blocker atenolol at optimal concentrations of laccase of 0.5 U mL^−1^ and mediator of 500 μM.^81^ Obleser *et al.*^25^ determined important parameters for oxidation reaction of anis alcohol to aldehyde using *T. versicolor* laccase/TEMPO system. The authors showed that the minimum effective laccase concentrations *>*0.25 U mL^−1^ with mediator : substrate ratio 0.3 equiv. In our case, laccase concentration of 0.12 U mL^−1^ is sufficient for successful oxidation of IC when loading the mediator 500 μM.

### 3.9 Effect of CuCl_2_, Cu(NO_3_)_2_ and CuSO_4_ on the laccase activity

Copper ions play a very important role in the processes of biooxidation involving laccases, because of Cu is located in the active center of laccase. It is known that the addition of copper to the nutrient medium stimulates the production of laccase,^82,83^ affects of the laccase activity and the rate of biodegradation of oxidizable compounds.^84,85^

**Fig. 10:**
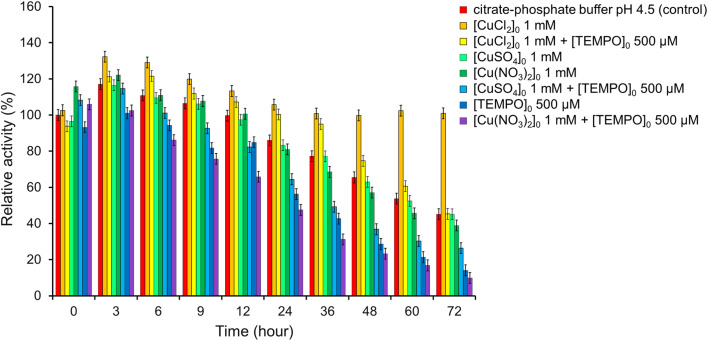
Impact of CuCl_2_, Cu(NO_3_)_2_, CuSO_4_ and TEMPO on the laccase activity. The error bars represent the standard deviation of triplicate samples.

To determine the effect of copper ions on the activity of *T. versicolor* laccase, the enzyme was incubated in a buffer solution in the presence of Cu(NO_3_)_2_, CuCl_2_ or CuSO_4_ at a concentration of 1 mM (Fig. 10, S3–S8, Tables S2–S7†) with and without TEMPO. It was found that laccase activity did not differ from that in a buffer solution in the presence of CuSO_4_ without TEMPO: laccase remained stable for 72 h. Cu(NO_3_)_2_ also did not affect the stability of laccase for 24 h. However, Cu(NO_3_)_2_ reduced the enzyme activity by 6.3% compared to the control sample with an increase in the incubation time to 72 h. The negative effect of Cu(NO_3_)_2_ was especially pronounced in the presence of a mediator. Catalytic system Cu(NO_3_)_2_ with TEMPO are effectiveness used for oxidation organic compounds,^86^ but it does not oxidase of ABTS in the our conditions. The residual activity of laccase was less than in the presence of only mediator by 19.1% after 12 h and 4.2% after 72 h. At the same time, CuSO_4_ had a positive effect on the laccase activity in the presence of TEMPO. It can be seen from Fig. 10 the residual activity of the enzyme after 72 h was 26.3%, while in the presence of only the mediator it was 14.1%, and in the presence of Cu(NO_3_)_2_ and the mediator it was 9.9%. These results clearly show the positive effect of CuSO_4_ as an inducer of laccase activity.

CuCl_2_ showed the best result. When salt is added to the laccase buffer solution, an increase in enzyme activity is observed up to 132% after 3 h and 129% after 6 h. The activity then slowly decreases to 106% after 24 h and remains stable even after 72 h of incubation (101%). CuCl_2_ significantly slows down the mediator inactivation of laccase. As can be seen from Fig. 10, laccase remains stable in the presence of TEMPO for 36 h of incubation, while the activity of the enzyme is higher than in the control sample by 18%. Then there is a gradual decrease in laccase activity to 45% after 72 h. These results clearly show the positive effect of CuCl_2_ as an inducer of laccase activity.

The influence of metal cations on laccase activity has been studied in numerous works.^71–73,82–85^ As for anions, there is very little information available on this matter.^87^ However, it is known that Cu(NO_3_)_2_ in combination with aminooxy radicals^86,88^ or their precursors,^89^ successfully catalyzes the oxidation of organic compounds with molecular oxygen. But it is obvious that Cu(NO_3_)_2_ negatively affects the *T. versicolor*. It is not possible to determine exactly the mechanism of the effect of anions on the enzyme based on the obtained results. However, it can be assumed that the enzyme inactivation occurs because of a specific interaction of nitrates with charged amino acid residues in the active site, which causes a change in the conformation of the enzyme with damage to the catalytic site.

## 4 Conclusions

In this work, we studied the main physicochemical parameters, such as pH, initial dye, and mediator concentrations to achieve maximum IC decolorization with the *T. versicolor* laccase/TEMPO/O_2_ biocatalytic system. The above results showed that IC decolorization occurs in a short time and that LMS can be reused for batch reactions. The system demonstrated 100% IC decolorization within 5 hours in the presence of dye bath salts and high IC concentration. Three major metabolites of IC biodegradation were identified using HPLC analysis coupled with UV spectroscopy. The study evaluated the long-term stability of the *T. versicolor* laccase in the presence of dye, mediator, and copper. Laccase is inactivated in the presence of TEMPO, which significantly limits the possibilities of LMS. Response surface plot of laccase relative activity from the initial TEMPO concentration over time was built. The optimal conditions for maintaining a sufficient level of enzymatic activity have been determined. A positive effect of CuCl_2_ and CuSO_4_ on the stability of laccase has been established, and Cu(NO_3_)_2_, on the contrary, inactivates the enzyme. The system under study can be used for complex wastewater treatment, since it is quite versatile and capable of oxidizing a wide range of stable organic compounds. The results obtained can be used as a guide for understanding the catalytic activity of the *T. versicolor* laccase/TEMPO/O_2_ system and optimizing processes involving it.

## Author contributions

Iryna O. Hordieieva: investigation, visualization, supervision, formal analysis, writing – original draft. Olga V. Kushch: writing – review & editing, validation, conceptualization, supervision. Tetiana O. Hordieieva: investigation, formal analysis. Serhii I. Sirobaba: investigation, formal analysis. Mykhailo O. Kompanets: investigation, validation, formal analysis. Victor M. Anishchenko: investigation, formal analysis. Alexander N. Shendrik: conceptualization, methodology, writing – original draft.

## Conflicts of interest

The authors declare that they have no known competing financial interests or personal relationships that could have appeared to influence the work reported in this paper.

## Supplementary Material

RA-013-D3RA03107A-s001
