# Supplementary material for: Eco-friendly TEMPO/laccase/O2 biocatalytic system for degradation of Indigo Carmine: operative conditions and laccase inactivation
Source: RSC Adv. 2023 Jul 11;13(30):20737–47. doi: 10.1039/d3ra03107a (PMC10334265; doi:10.1039/d3ra03107a)
Supplement: RA-013-D3RA03107A-s001 [file RA-013-D3RA03107A-s001.pdf]

Electronic Supplementary Material (ESI) for RSC Advances.
This journal is © The Royal Society of Chemistry 2023

Eco-friendly TEMPO/Laccase/O_2 biocatalytic system for degradation of Indigo Carmine: operative conditions and laccase inactivation

Iryna O. Hordieieva[a,b*], *Olga V. Kushch*[a,b*], *Tetiana O. Hordieieva*[a], *Serhii I. Sirobaba*[a], *Mykhailo O. Kompanets*[b], *Victor M. Anishchenko*[b], *Alexander N. Shendrik*[a]

[a]*Faculty of Chemistry, Biology and Biotechnologies, Vasyl' Stus Donetsk National University, Vinnytsia, 21021 Ukraine*
[b]*L.M. Litvinenko Institute of Physico-Organic Chemistry and Coal Chemistry, National Academy of Sciences of Ukraine, Kyiv, 02660 Ukraine*
*Corresponding authors:
Irina O. Hordieieva [a,b*], Olga V. Kushch [a,b*]
Email: i.hordieeva@donnu.edu.ua, kusch.o@donnu.edu.ua

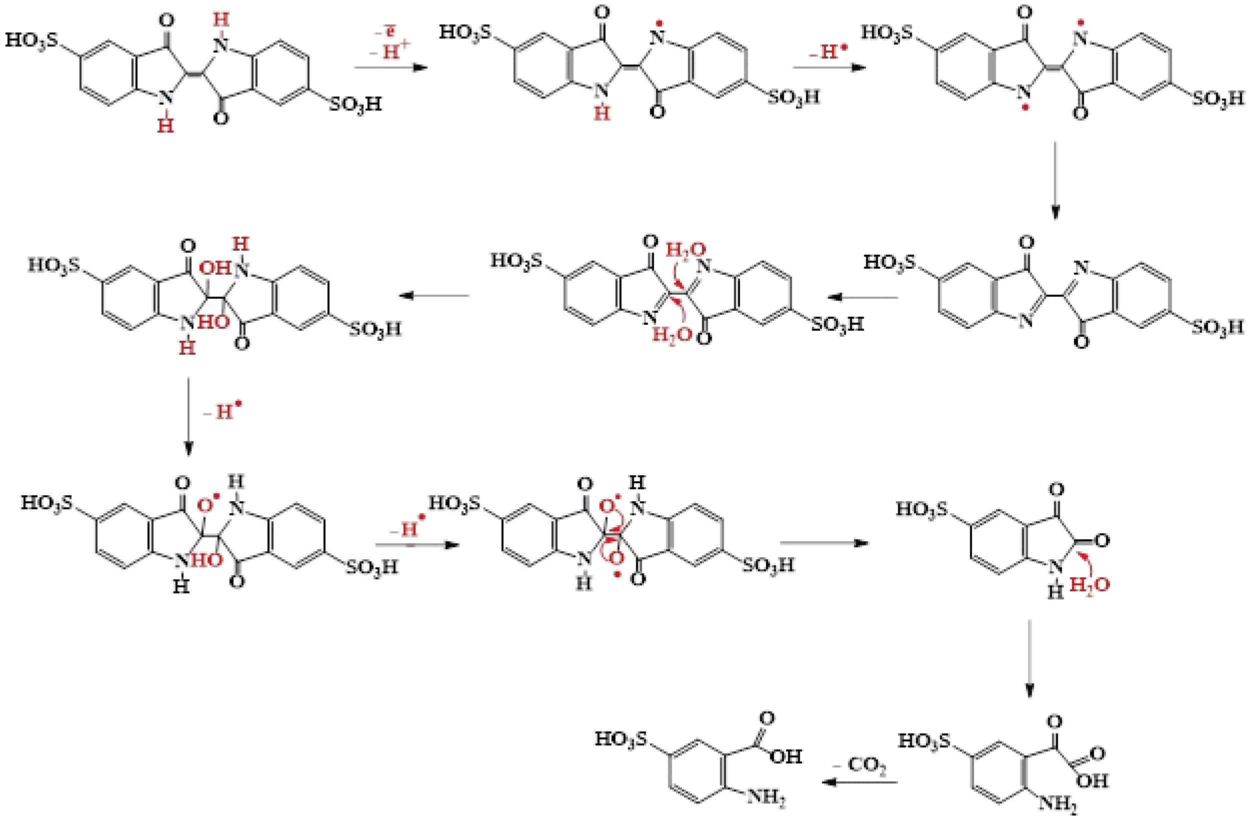

Scheme S1. Possible mechanism of Indigo Carmine degradation

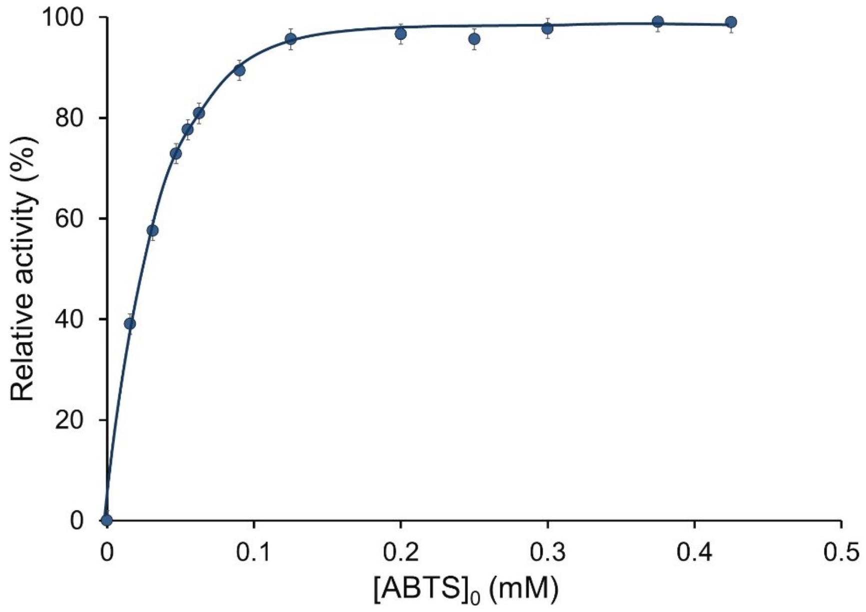

Fig. S1. Effect of ABTS concentration on the *T. versicolor* laccase relative activity at $[laccase]_0 = 6.0 \cdot 10^{-3}$ U·mL^{-1} in the citrate-phosphate buffer at pH 4.5 at 35 °C

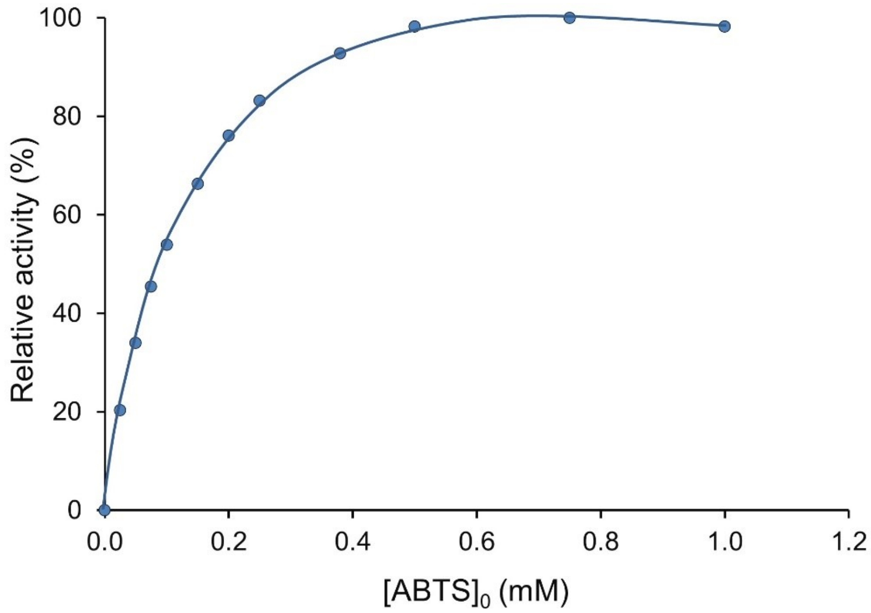

Fig. S2. Effect of ABTS concentration on the *T. versicolor* laccase relative activity at $[laccase]_0 = 0.12$ U·mL^{-1} in the citrate-phosphate buffer at pH 4.5 at 35 °C

Table S1

The effect of initial TEMPO concentration and time on laccase relative activity. $[laccase]_0 = 0.12$ $U \cdot mL^{-1}$ in the citrate-phosphate buffer at pH 4.5 at 35 °C

t, hour	$[TEMPO]_0$, mM							
	0	0.1	0.3	0.5	0.8	1.0	1.3	1.5
	Relative activity, %							
0.0	100.00	100.0	92.7	93.2	96.4	83.9	87.0	90.1
3.0	117.10	94.3	87.5	101.0	99.0	98.4	104.2	94.3
6.0	110.81	90.6	91.1	94.3	88.5	84.4	82.3	79.7
9.0	106.45	83.9	88.0	81.8	83.9	84.4	79.7	71.9
12.0	99.68	85.4	83.9	84.9	82.3	83.9	75.5	69.3
24.0	85.97	59.4	56.8	56.3	51.0	47.9	41.7	34.4
36.0	77.26	47.9	44.3	42.7	36.5	34.9	27.1	25.0
48.0	65.5	32.3	27.1	28.6	21.4	19.3	12.5	12.0
60.0	56.0	26.0	20.3	21.4	15.1	13.5	7.8	6.3
72.0	45.2	19.8	14.6	14.1	9.9	7.3	3.6	2.6
84.0	37.6	15.6	10.4	10.9	6.8	5.2	2.6	2.1
96.0	31.6	13.0	7.8	8.3	4.2	3.1	2.6	0.0

Table S2

The effect of initial TEMPO and $CuSO_4$ concentrations and time on laccase relative activity. $[laccase]_0$ = 0.12 U·mL^{-1}, $[TEMPO]_0$ = 0.5 mM in the citrate-phosphate buffer at pH 4.5 at 35 °C

t, hour	$[CuSO_4]_0$, mM				
	0	0.5	1	5	10
	Relative activity, %				
0.0	100.0	106.8	108.2	104.9	100.2
3.0	117.1	110.9	114.7	101.2	89.6
6.0	110.8	99.7	101.1	93.6	80.9
9.0	106.5	87.9	92.6	84.2	70.6
12.0	99.7	78.5	82.3	72.4	63.1
24.0	86.0	59.8	64.5	56.1	46.3
36.0	77.3	50.0	49.3	44.3	36.8
48.0	65.5	38.2	36.8	33.6	28.0
60.0	53.7	30.3	30.3	27.7	22.9
72.0	45.2	25.4	26.3	23.2	19.1

Table S3

The effect of initial TEMPO and $Cu(NO_3)_2$ concentrations and time on laccase relative activity. $[laccase]_0$ = 0.12 U·mL^{-1}, $[TEMPO]_0$ = 0.5 mM in the citrate-phosphate buffer at pH 4.5 at 35 °C

t, hour	$[Cu(NO_3)_2]_0$, mM				
	0	0.5	1	5	10
	Relative activity, %				
0.0	100.0	98.5	105.9	86.1	85.1
3.0	117.1	93.6	102.5	82.2	90.1
6.0	110.8	81.2	86.1	69.8	69.3
9.0	106.5	69.8	75.7	60.4	55.0
12.0	99.7	61.9	65.8	54.5	42.6
24.0	86.0	41.1	47.5	38.1	24.3
36.0	77.3	30.2	31.2	26.2	17.8
48.0	65.5	21.3	23.3	19.8	11.4
60.0	56.0	14.9	16.8	13.9	8.4
72.0	45.2	10.4	9.9	9.4	5.9

Table S4

The effect of initial $Cu(NO_3)_2$ concentration and time on laccase relative activity. $[laccase]_0$ = 0.12 U·mL^{-1} in the citrate-phosphate buffer at pH 4.5 at 35 °C

t, hour	$[Cu(NO_3)_2]_0$, mM				
	0	0.5	1	5	10
	Relative activity, %				
0.0	100.00	106.5	115.8	119.8	123.4
3.0	117.10	117.9	122.1	125.5	122.6
6.0	110.81	110.3	111.0	110.6	104.4
9.0	106.45	100.5	107.7	102.9	93.1
12.0	99.68	99.0	100.6	97.4	81.5
24.0	85.97	77.7	81.0	73.5	55.5
36.0	77.26	64.2	68.5	59.2	42.6
48.0	65.5	48.2	57.1	42.4	29.0
60.0	55.8	33.9	45.0	27.3	15.9
72.0	45.2	36.1	38.9	18.9	11.9

Table S5

The effect of initial $CuSO_4$ concentration and time on laccase relative activity. $[laccase]_0$ = 0.12 U·mL^{-1} in the citrate-phosphate buffer at pH 4.5 at 35 °C

t, hour	$[CuSO_4]_0$, mM				
	0	0.5	1	5	10
	Relative activity, %				
0.0	100.0	96.5	102.1	110.1	113.7
3.0	117.1	116.5	114.7	114.6	117.9
6.0	110.8	109.5	106.5	98.5	103.6
9.0	106.5	106.2	105.9	93.1	91.1
12.0	99.7	97.4	98.7	85.8	82.3
24.0	86.0	83.2	86.6	72.2	63.5
36.0	77.3	77.2	80.5	58.1	54.8
48.0	65.5	63.0	69.8	47.7	45.5
60.0	53.7	52.6	65.6	43.0	42.7
72.0	45.2	45.1	59.8	31.0	28.8

Table S6

The effect of initial $CuCl_2$ concentration and time on laccase relative activity. $[laccase]_0$ = 0.12 U·mL^{-1} in the citrate-phosphate buffer at pH 4.5 at 35 °C

t, hour	$[CuCl_2]_0$, mM					
	0	0.5	1	5	10	50
	Relative activity, %					
0.0	100.0	87.0	81.1	96.1	102.6	45.2
3.0	117.2	128.0	117.5	129.5	132.3	24.6
6.0	116.2	126.1	118.2	129.3	129.0	16.1
9.0	116.4	121.6	114.9	121.1	119.8	10.3
12.0	111.0	117.9	111.8	120.0	113.3	9.7
24.0	80.0	89.0	89.5	123.0	105.7	9.1
36.0	72.8	80.2	80.5	125.2	100.8	7.5
48.0	60.3	68.7	68.5	120.3	99.8	3.3
60.0	47.9	63.9	60.2	114.1	102.5	2.3
72.0	40.0	58.7	73.1	107.9	101.0	0.5

Table S7

The effect of initial TEMPO and $CuCl_2$ concentrations and time on laccase relative activity. $[laccase]_0$ = 0.12 U·mL^{-1}, $[TEMPO]_0$ = 0.5 mM in the citrate-phosphate buffer at pH 4.5 at 35 °C

t, hour	$[CuCl_2]_0$, mM					
	0	0.5	1	5	10	50
	Relative activity, %					
0.0	100.0	83.8	93.8	99.4	105.3	90.6
3.0	105.9	120.6	121.3	121.9	125.9	103.8
6.0	112.8	123.1	121.6	129.7	131.6	102.6
9.0	111.0	106.6	111.9	103.4	120.6	100.0
12.0	109.1	101.3	107.2	100.9	110.3	97.5
24.0	103.1	83.4	100.3	105.6	114.1	92.5
36.0	97.5	67.8	95.0	95.9	103.4	85.3
48.0	74.4	41.3	74.7	76.3	78.4	66.6
60.0	65.9	30.0	60.6	60.9	67.8	55.6
72.0	51.6	20.0	45.3	40.9	55.0	47.5

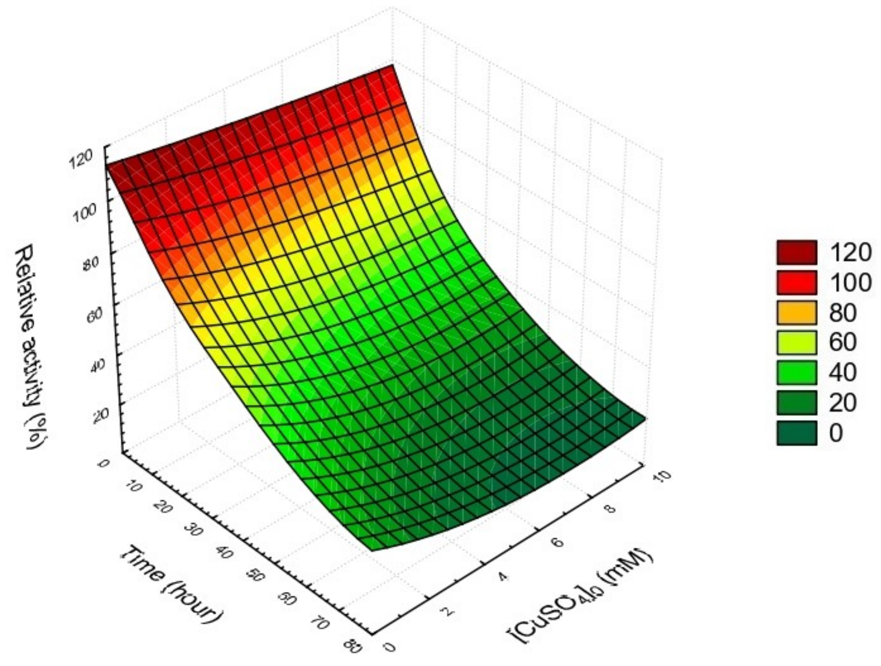

Fig. S3. 3D surface plot of the effect of initial TEMPO and $CuSO_4$ concentrations and time on laccase relative activity. $[laccase]_0 = 0.12$ U·mL^{-1}, $[TEMPO]_0 = 0.5$ mM in the citrate-phosphate buffer at pH 4.5 at 35 °C

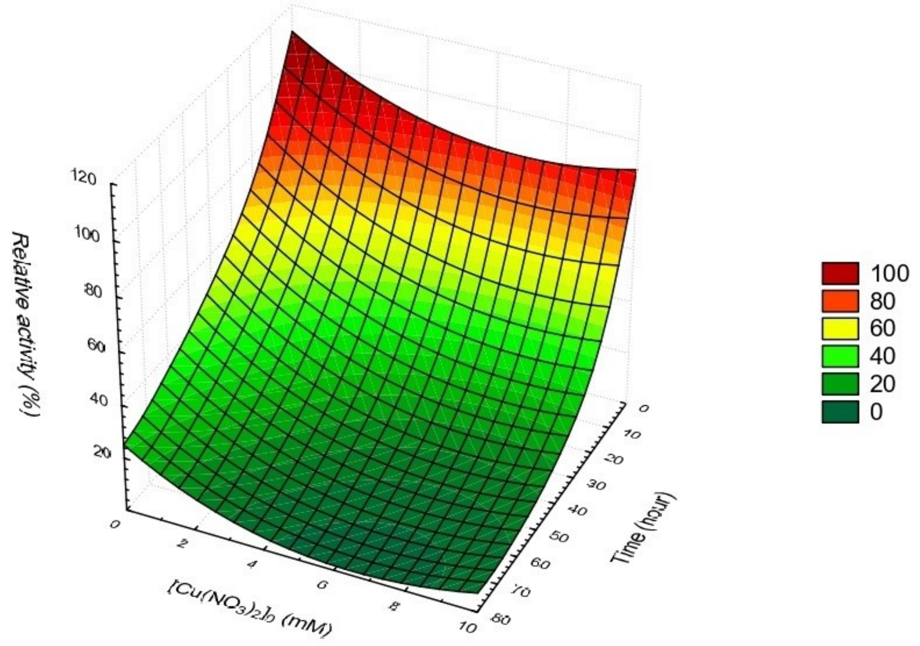

Fig. S4. 3D surface plot of the effect of initial TEMPO and $Cu(NO_3)_2$ concentrations and time on laccase relative activity. $[laccase]_0 = 0.12$ U·mL^{-1}, $[TEMPO]_0 = 0.5$ mM in the citrate-phosphate buffer at pH 4.5 at 35 °C

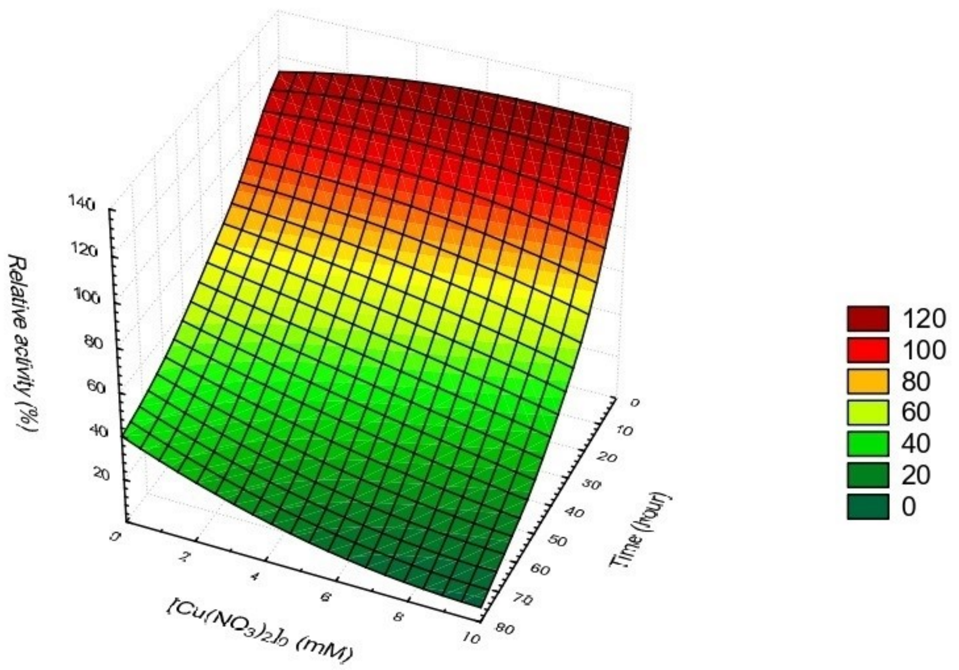

Fig. S5. 3D surface plot of the effect of initial $Cu(NO_3)_2$ concentration and time on laccase relative activity. $[laccase]_0 = 0.12$ U·mL^{-1} in the citrate-phosphate buffer at pH 4.5 at 35 °C

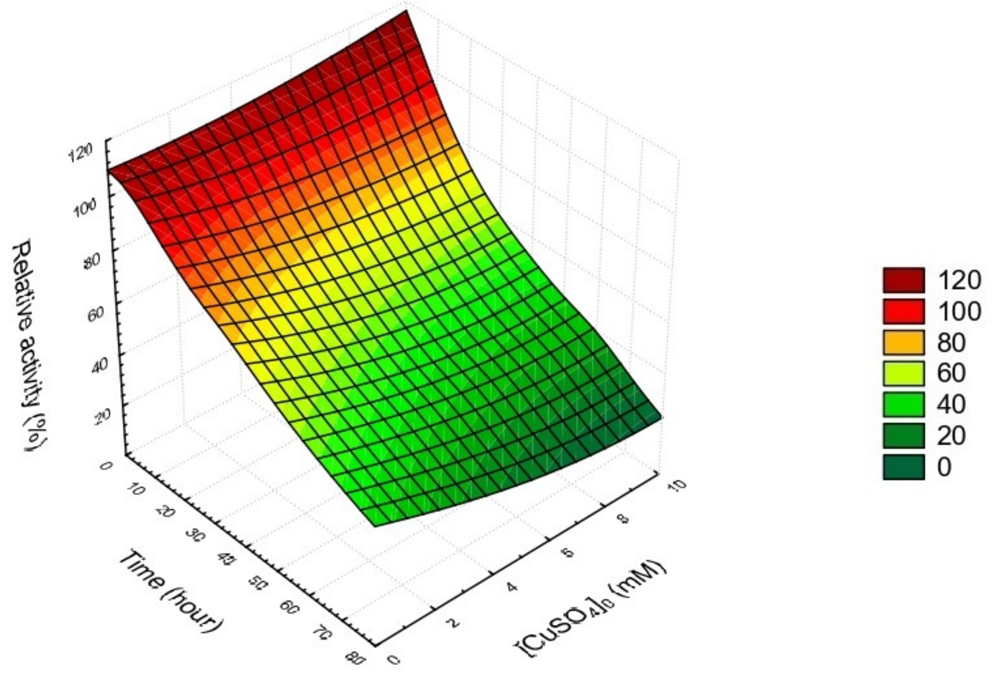

Fig. S6. 3D surface plot of the effect of initial $CuSO_4$ concentration and time on laccase relative activity. $[laccase]_0 = 0.12$ U·mL^{-1} in the citrate-phosphate buffer at pH 4.5 at 35 °C

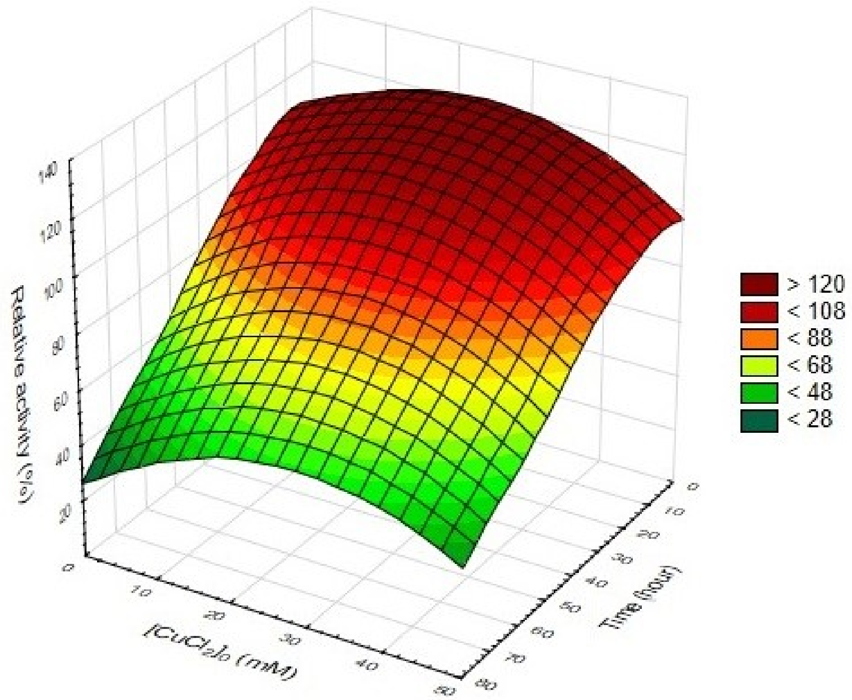

Fig. S7. 3D surface plot of the effect of initial TEMPO and $CuCl_2$ concentrations and time on laccase relative activity. $[laccase]_0$ = 0.12 U·mL^{-1}, $[TEMPO]_0$ = 0.5 mM in the citrate-phosphate buffer at pH 4.5 at 35 °C

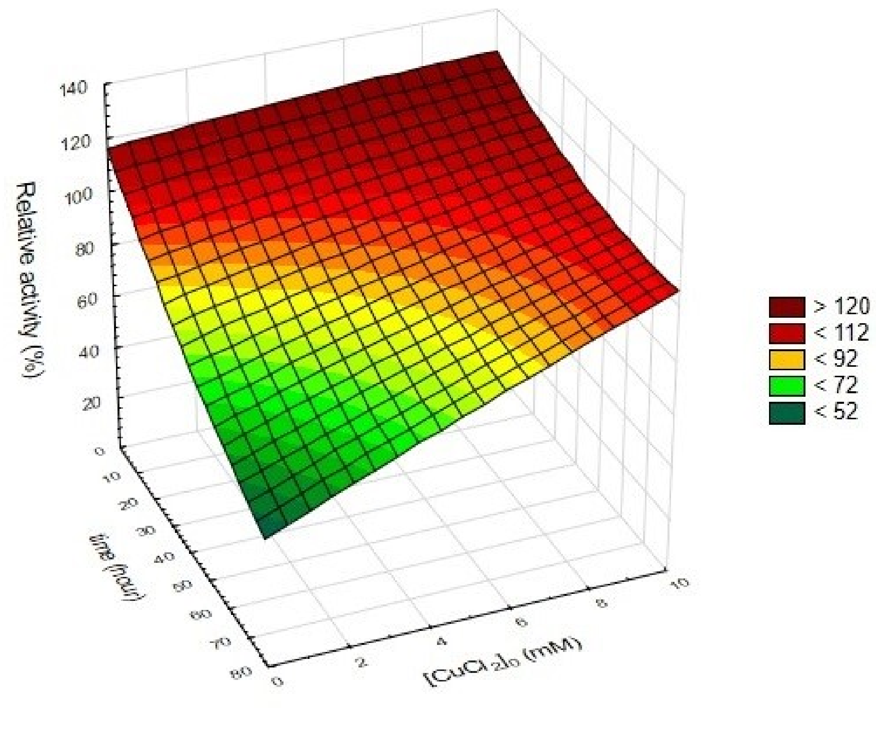

Fig. S8. 3D surface plot of the effect of initial $CuCl_2$ concentration and time on laccase relative activity. $[laccase]_0$ = 0.12 U·mL^{-1} in the citrate-phosphate buffer at pH 4.5 at 35 °C
